# (Seltene) infektiöse Hepatitiden als wichtige Differenzialdiagnose der unklaren Hepatopathie

**DOI:** 10.1007/s00292-022-01167-0

**Published:** 2022-12-01

**Authors:** Michael Wührl, Marc Ringelhan, Ursula Ehmer, Jochen Schneider, Juliane Kager, Tobias Lahmer, Anna Schneider, Wilko Weichert, Carolin Mogler

**Affiliations:** 1 Institut für allgemeine Pathologie und pathologische Anatomie, Technische Universität München, Trogerstr. 18, 81675 München, Deutschland; 2 Klinik und Poliklinik für Innere Medizin II, Klinikum rechts der Isar, Technische Universität München, München, Deutschland; 3 Klinik und Poliklinik für Frauenheilkunde, Klinikum rechts der Isar, Technische Universität München, München, Deutschland

**Keywords:** Unklares Leberversagen, Leberbiopsie, Infektiologie, Hepatologie, Hepatitis, Liver failure, Infectious disease medicine, Hepatitis, Liver diseases

## Abstract

**Hintergrund:**

Die (transjuguläre) Leberbiopsie stellt einen essenziellen diagnostischen Baustein im Diagnosealgorithmus der unklaren Hepatopathie bzw. des akuten Leberversagens dar. Die Beurteilung und Auswertung erfordert eine eng verzahnte Zusammenarbeit zwischen Klinikern und Pathologen, die klinischen Umstände und die oftmals lebensbedrohlichen Komplikationen des akuten Leberversagens machen eine zeitnahe Diagnosefindung notwendig. Insbesondere seltenere infektiöse Hepatitiden werden mitunter im klinischen Kontext nicht oder nur verzögert diagnostiziert, sodass dem Pathologen hier eine maßgebliche Rolle zu Teil wird und der Patient so zeitnah einer zielgerichteten Therapie zugeführt werden kann.

**Ziel der Arbeit (Fragestellung):**

In der Leberbiopsie seltene aber teils sehr prägnante, da unbehandelt mitunter tödlich verlaufende infektiöse Ursachen des unklaren Leberversagens werden vorgestellt.

**Material und Methoden:**

Retrospektive Fälle von Leberbiopsien bei unklarer Hepatopathie und serologisch bzw. molekularbiologisch bestätigter Infektion wurden aus der internen Falldatenbank des Instituts für Pathologie der TU München ausgewählt und hinsichtlich der histomorphologischen Diagnosekriterien der jeweiligen infektiösen Erkrankungen aufgearbeitet.

**Ergebnisse und Diskussion:**

Im Untersuchungsgut wurden neben den klassischen viralen Hepatitiden auch seltene infektiöse Hepatitiden, ausgelöst durch Adenoviren, Herpes-simplex-Virus und Rickettsien, identifiziert. Diese Erkrankungen sind selten, aber mitunter lebensbedrohlich. Durch die Kenntnisse der histomorphologischen Veränderungen lassen sich frühzeitig die weitere Diagnostik und Therapie bahnen und somit unter Umständen lebensbedrohliche Verläufe abwenden.

## Abstract (en)

**Background:**

Liver biopsies to determine the cause of unclear hepatopathy and acute liver failure represent a diagnostic hallmark and require close cooperation between clinicians and pathologists. The commonly acute presentation of hepatic dysfunction warrants a rapid diagnosis. Infectious causes of hepatitis may be identified by the pathologist, supporting further diagnostic and therapeutic steps.

**Objectives:**

Rare infectious causes of hepatic dysfunction with distinct histomorphological features are presented.

**Materials and methods:**

Retrospective cases of liver biopsies for evaluation of hepatic dysfunction with infection confirmed by laboratory tests were selected from the archive of the institute of pathology of TUM and evaluated for morphologic diagnostic criteria.

**Results and conclusions:**

Infections with adenovirus, Herpes simplex virus, Hepatitis A virus, and coxiella burnettii are rare findings in liver core biopsies but able to cause hepatic dysfunction that present with distinct histomorphologic alterations that can contribute to the identification of the causative agent. Rare infectious causes should be considered in any patient presenting with hepatic dysfunction of unknown etiology. Knowledge of the histomorphologic criteria by the pathologist is crucial to initiate further diagnostic testing and treatment.

Trotz aller Fortschritte im Bereich der Laborchemie und Bildgebung stellt die Leberbiopsie nach wie vor ein wichtiges diagnostisches Werkzeug zur ätiologischen Klärung der unklaren Hepatopathie bzw. des akuten Leberversagens dar [31]. Die geringe Materialmenge bei oft gleichzeitig hoher Anzahl an Differenzialdiagnosen und häufig akut verlaufenden Krankheitsbildern mit drohendem akutem Leberversagen stellen eine Herausforderung an die Pathologie dar [17].

Durch eine zeitnahe Einordnung können weitere diagnostische und therapeutische Schritte veranlasst werden. Umso wichtiger sind daher die Kenntnisse auch über seltene, infektiöse Lebererkrankungen und deren typische (und weniger typische) histomorphologischen Veränderungen, die konkrete Hinweise zur Ätiologie im Befundbericht an die klinischen Kollegen erlauben. Infektionen sind nach der medikamentös-toxischen Leberschädigung (z. B. Paracetamolvergiftung) die zweithäufigste Ursache des akuten Leberversagens [17]. Der Großteil akuter Leberinfektionen wird hierbei durch Hepatitis-A- und Hepatitis-B-Viren verursacht [5]. Andere Erreger sind lediglich für einen Bruchteil der Infektionen, etwa im einstelligen Prozentbereich verantwortlich [32], können aber gerade im immunsupprimierten Patienten schwere Verläufe verursachen [1]. Im Folgenden werden an retrospektiven Fallvignetten in der Leberbiopsie seltene, infektiöse Differenzialdiagnosen bei unklarer Hepatopathie präsentiert.

Mittels retrospektiver Datenbankanalyse wurde der Leberbiopsieeingang am Institut für Pathologie der TU München der Jahre 2017–2022 (5 Jahre) ausgewertet im Hinblick auf histomorphologisch suspizierte und klinisch (serologisch oder molekular) bestätigte Leberinfektionen mit dem klinischen Bild einer unklaren Hepatopathie. Hierbei wurden insgesamt 7 Fälle seltener infektiöser Ursachen identifiziert. In 6 Fällen handelte es sich um virale Erkrankungen (2 Fälle Adenovirus, 2 Fälle Herpes-simplex-Virus sowie jeweils ein Fall Hepatitis-A- bzw. Hepatitis-E-Virus), in einem Fall um eine bakterielle Infektion (Rickettsien). Allen Fällen gemeinsam war ein progredienter klinischer Verlauf mit teils Progress zum akuten Leberversagen bzw. in einem Fall zur Zirrhose, 4 Fälle endeten im Exitus letalis (1 × Adenovirus, 1 × Herpes-simplex-Virus, 2 × Grunderkrankung). Das mediane Alter der Patienten bei Erkrankung lag bei 50 Jahren (38–59 Jahre), erkrankt waren 5 Männer und 2 Frauen. In 4 Fällen lag eine Immunsuppression vor (Zustand nach Stammzelltransplantation, hämatologische Grunderkrankung), in einem Fall eine Hysterektomie mit Scheidenstumpfinsuffizienz und in 2 Fällen keine relevanten Grunderkrankungen.

Eine Übersicht über alle hier vorgestellte Fälle finden sich in Tab. 1.

**Table 1:** 

Fall	Geschlecht	Alter	Grunderkrankung/relevantes aus Anamnese und klinischer Untersuchung	Erreger/Nachweistechnik	Verlauf
1	Männlich	54	Multiples Myelom; Z. n. homologer Stammzelltransplantation	Adenovirus PCR	Verstorben
2	Männlich	56	Rezidiv diffus großzelliges B‑Zell-Lymphom	Adenovirus PCR	Ausgeheilt, im Verlauf verstorben
3	Weiblich	40	Hysterektomie vor 3 Monaten, aktuell Scheidenstumpfinsuffizienz	HSV Serologie (IgM/IgG) negativ, PCR aus Serumrückstellprobe	Verstorben
4	Weiblich	45	Z. n. Nierentransplantation	HSV Serologie/PCR	Ausgeheilt
5	Männlich	38	Keine relevanten bekannt	*C. burnettii* Serologie	Ausgeheilt
6	Männlich	59	Urlaub in Sri Lanka vor 2 Monaten	HAV Serologie	Listung zur Transplantation, im Verlauf dann spontane Befundbesserung
7	Männlich	50	Z. n. Stammzelltransplantation bei follikulärem Lymphom	HEV PCR	Chronifizierung der Hepatitis E unter Immunsuppression, im Verlauf verstorben

*HAV* Hepatitis A Virus, *HEV* Hepatitis E Virus, *HSV* Herpes simplex Virus, *PCR* polymerase chain reaction

## Adenoviren

In der Datenbankanalyse konnten 2 Fälle von Adenovirusinfektionen identifiziert werden.

### Fall 1

**Figure 1:**
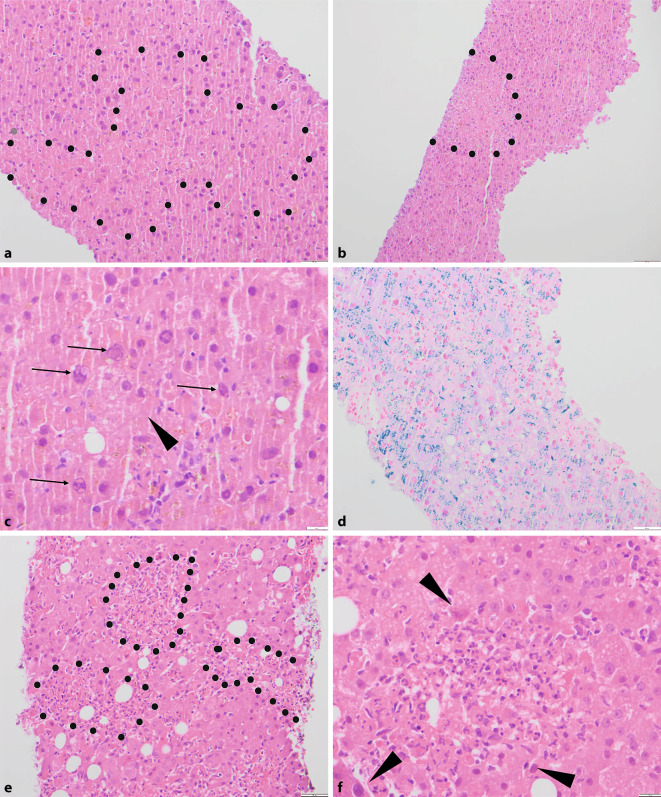

Ein 55-jähriger männlicher Patient mit Zustand nach homologer Stammzelltransplantation wurde bei akutem Leberversagen stationär aufgenommen. In der Stanzbiopsie zeigte sich ein ausgeprägtes hepatitisches Schädigungsbild (Abb. 1a) mit vergleichsweise geringer entzündlicher Aktivität in den Portalfeldern, im Parenchym aber ausgedehnten diffusen, teils konfluierenden multifokalen (nichtzonalen) hepatozellulären Nekrosen ohne signifikante entzündliche Begleitreaktion (Abb. 1b). Angrenzend an die Nekrosen imponierten die Hepatozytenkerne mit an den Rand gedrängtem basophilem Chromatin und zentralem eosinophilem, trübem Einschluss (Abb. 1c). Zusätzlich zeigte sich in der Berliner-Blau-Reaktion passend zu wiederholten Bluttransfusionen bei Zustand nach Stammzelltransplantation eine massive Eisenüberladung der Leber (Abb. 1d). Der Patient verstarb wenige Tage später im Multiorganversagen.

### Fall 2

Ein 56-jähriger männlicher Patient wurde unter laufender Chemotherapie bei Rezidiv eines diffus-großzelligen B‑Zell-Lymphoms aufgrund auffälliger Infektparameter in der hausärztlichen Blutkontrolle stationär aufgenommen. Im Verlauf kam es hier zu steigenden Transaminasewerten, woraufhin bei unklarer Ursache eine transjuguläre Leberbiopsie durchgeführt wurde. Histologisch zeigten sich in diesem Fall ebenfalls diffus verteilte, flächige Nekrosen des Leberparenchyms (Abb. 1e). Angrenzend an die Nekroseareale zeigten die Hepatozyten ähnlich wie im vorherigen Fall Kerneinschlüsse, wenn auch etwas weniger ausgeprägt und überwiegend glasig eosinophil (Abb. 1f). Darüber hinaus zeigte sich ein ausgeprägtes, granulozytär dominiertes Infiltrat in dichter Assoziation mit den Nekrosen als Zeichen bereits stattfindender Abräumreaktion (Abb. 1f). Unter eingeleiteter Therapie kam es zwar zur Befundbesserung, der Patient verstarb dann aber im Verlauf.

## Herpes-simplex-Virus (HSV)

In der Datenbankanalyse konnten 2 Fälle von Herpes-simplex-Virus-Infektionen identifiziert werden.

### Fall 1

**Figure 2:**
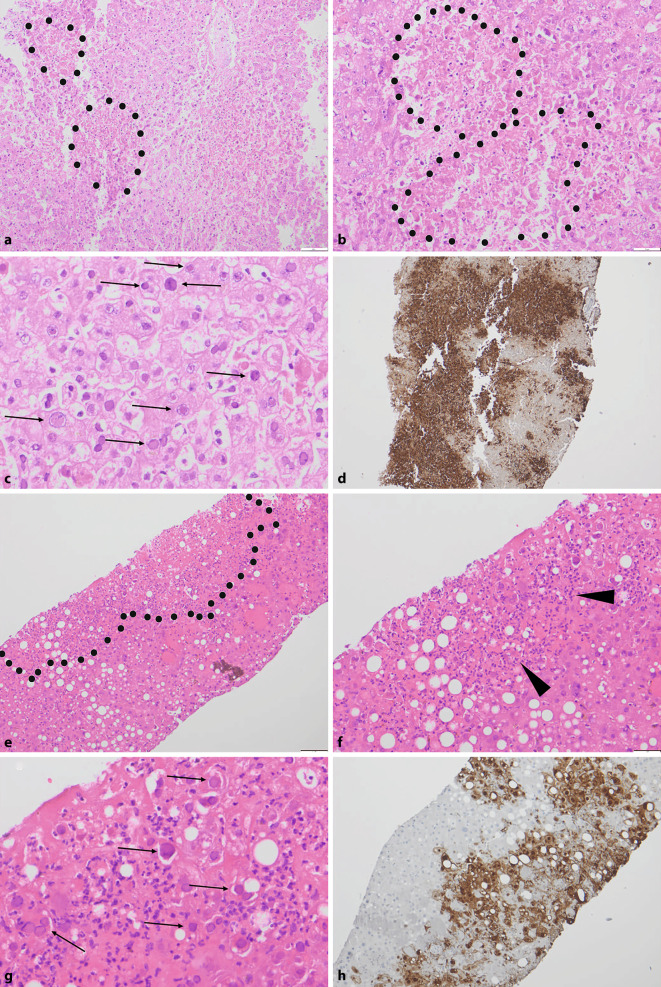

Eine 40-jährige weibliche Patientin wurde mittels Rettungsdienst in der Notaufnahme vorstellig. Als relevante Voroperation war in einem auswärtigen Krankenhaus vor 3 Monaten eine Hysterektomie durchgeführt worden. In der Bildgebung zeigten sich Abszesse im kleinen Becken, die operativ ausgeräumt wurden. Postoperativ kam es unter Antibiose zunächst zu Beschwerdebesserung mit anschließendem, plötzlichem, starkem Transaminasenanstieg. Es erfolgte daher bei unklarer Klinik und drohendem Leberversagen eine Stanzbiopsie der Leber. Histologisch zeigten sich diffus auf alle Zonen verteilte Nekrosen mit teils flächiger Konfluierung (Abb. 2a,b). Im Randbereich der Nekrosen zeigten sich eine auffällige Ballonierung der Hepatozyten und hier insbesondere deutlich trübe bis milchglasartige Kerne mit Chromatinkondensation im Sinne viraler Einschlusskörperchen (Abb. 1c). In der Herpes-simplex-Virus(HSV)-Immunhistochemie zeigte sich eine diffuse Anfärbung der befallenen Hepatozyten (Abb. 1d). Bei negativer Serologie (IgM und IgG), aber immunhistochemisch eindeutigem Befund erfolgte zusätzlich noch der DNA-Nachweis von HSV‑1 mittels PCR am Paraffinmaterial. In einer Serumrückstellprobe konnte auch klinisch HSV nachgewiesen werden. Eine umgehend eingeleitete antivirale Therapie war bei bereits fortgeschrittenem akutem Leberversagen nicht mehr wirksam. Die Patientin verstarb kurze Zeit später im septischen Schock. In der anschließenden Obduktion in domo konnte das Leberversagen und das konsekutive septische Multiorganversagen durch Infektion mit HSV bestätigt werden.

### Fall 2

Eine 45-jährige Patientin mit Z. n. Nierentransplantation war wegen steigender Transaminasenwerte kurze Zeit nach Umstellung der Immunsuppression als Reaktion auf eine mögliche Abstoßung stationär aufgenommen worden. Histologisch zeigte sich bei der ersten Biopsie eine Hepatozytenballonierung, Kupffer-Zell-Aktivierung sowie diffus verteilte und teils flächig konfluierende Gewebenekrosen mit angrenzenden viralen Einschlusskörperchen in Form glasiger, eosinophiler Kerne mit an den Rand gedrängtem Chromatin (Abb. 2e–g). Immunhistochemisch war eine deutliche Positivität für HSV erkennbar (Abb. 2h). Nach sofortiger Einleitung einer antiviralen Therapie kam es zu einer langsamen Besserung der Transaminasen mit kompletter Ausheilung im längerfristigen Verlauf.

## *Coxiella burnetii*

### Fall 1

**Figure 3:**
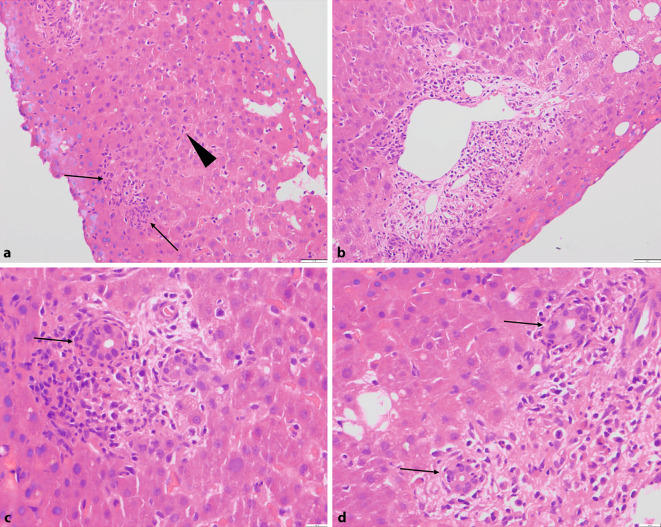

Ein 38-jähriger männlicher Patient wurde mit unklarer Transaminasenerhöhung stationär aufgenommen. Klinisch dominierten ein abrupt einsetzendes Fieber, starke Abgeschlagenheit mit Myalgien sowie trockenem Husten. Der Patient war Mitarbeiter im Außendienst in der Tierfuttermittelindustrie. Serologisch zeigte sich am Tag der Leberbiopsie der Phase-1-IgA-ELISA positiv für *Coxiella burnetti*. Die übrigen Phase-1-IgG/IgM- bzw. Phase-2-IgG/IgM-ELISAs waren negativ bzw. unter dem Grenzwert. Der Befund wurde durch ein Referenzlabor bestätigt. Es wurde der Verdacht auf eine zurückliegende Infektion ohne Hinweis auf Chronifizierung geäußert. Knapp 2 Monate später waren alle serologischen Untersuchungen auf *Coxiella burnettii* negativ. Histologisch zeigte die durchgeführte Leberbiopsie ein gemischtzelliges hepatitisch/cholangitisches Bild, zum einem mit entzündlichem Infiltrat in den Portalfeldern und im Parenchym verteilten Plasmazellen, Histiozyten, Lymphozyten, eosinophilen und neutrophilen Granulozyten (Abb. 3a,b) sowie zum anderem eine ausgeprägte floride Cholangitis (Abb. 3c,d). Im azinären Parenchym zeigten sich zudem einzelne Zelluntergänge, Entzündungszellen sowie geringe cholestatische Veränderungen. Fibrin-Ringgranulome konnten nicht eindeutig nachgewiesen werden. Unter antibiotischer Therapie konnte dann eine vollständige Ausheilung erreicht werden.

## Hepatitis-A-Virus

### Fall 1

**Figure 4:**
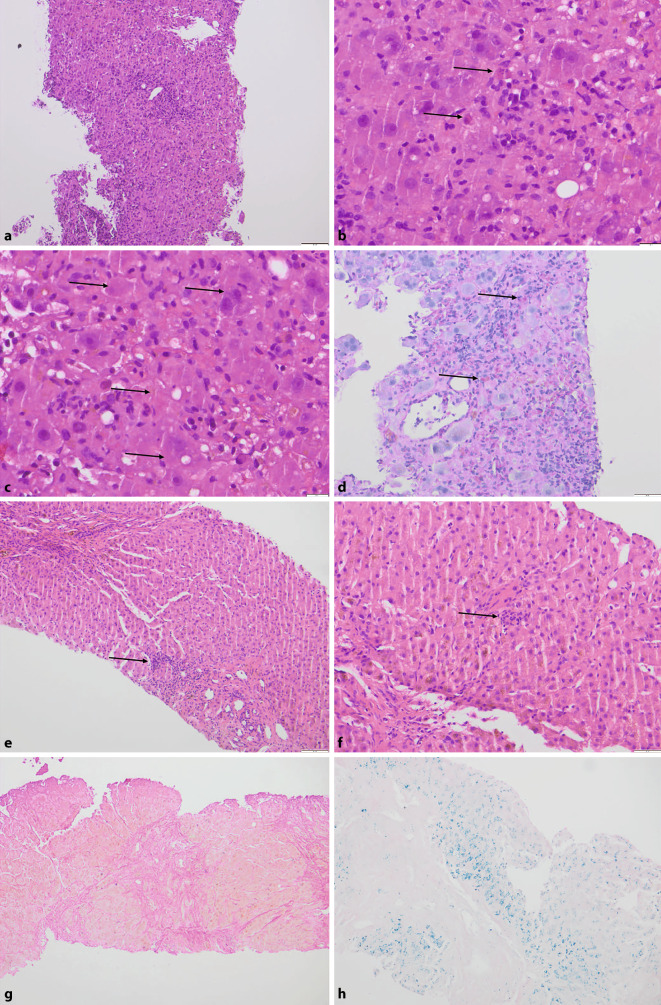

Ein 59 Jahre alter, aus Sri Lanka stammender und in Deutschland lebender männlicher Patient wurde beim Hausarzt mit Müdigkeit und beginnendem Ikterus vorstellig. Der letzte Aufenthalt in Sri Lanka lag gut 2 Monate zurück. Im Labor zeigte sich ein massiver Transaminasen- und Bilirubinanstieg, zudem eine eingeschränkte Gerinnung. Bei klinisch zunehmender Müdigkeit und Verdacht auf beginnende hepatische Enzephalopathie wurde eine Leberbiopsie in domo durchgeführt und der Patient an ein Transplantationszentrum weiterverlegt. In der Histologie zeigte sich ein akutes hepatitisches Schädigungsbild mit dichten lymphoplasmazellulären Infiltraten portal, im Parenchym sowie auch perivenulär (Abb. 4a), Apoptosen (Abb. 4b), Ballonierung der Hepatozyten, zum Teil Doppelkernigkeit (Abb. 4c) sowie mit nicht mehr ganz frischen, bereits entzündlich abgeräumten Einzelzell- und Gruppennekrosen (Abb. 4d). Im Verlauf wurde bei spontaner Befundbesserung von einer Listung zur Lebertransplantation abgesehen, die Infektion ist inzwischen ausgeheilt.

## Hepatitis-E-Virus

### Fall 1

Bei einem 50-jährigen Patient mit Zustand nach Stammzelltransplantation (bei follikulärem Lymphom) vor Jahren werden in einer Routineuntersuchung Ösophagusvarizen festgestellt und zur Abklärung eine Leberbiopsie durchgeführt. Beim Patienten war vor Jahren eine Hepatitis-E-Infektion diagnostiziert worden, die unter Immunsuppression zwar chronifizierte, in den letzten Untersuchungen aber nicht mehr nachgewiesen wurde (kein Nachweis von Hepatitis-E-Virus[HEV]-RNA, IgM und IgG negativ). Aktuell wurde erneut ein HEV-RNA-Nachweis im Blut erbracht und unter der Annahme einer erneuten Reaktivierung von HEV eine Therapie mit Ribavirin eingeleitet, was dazu führte, dass im Verlauf erneut HEV RNA nicht mehr nachweisbar war. In der Leberstanze zeigte sich ein unspezifisches schütteres, portalfeldassoziiertes Entzündungszellinfiltrat (Abb. 4e) mit lediglich einzelnen Entzündungszellfoci auch im Parenchym im Sinne eines milden hepatitischen Schädigungsbildes (Abb. 4f). Passend zu klinisch manifesten Ösophagusvarizen konnte histologisch eine Zirrhose nachgewiesen werden (Abb. 4g) in erster Linie als Folge der chronifizierten Hepatitis E, als Therapiefolge wurde zusätzlich eine Eisenüberladung beobachtet (Abb. 4h). Der Patient verstarb im weiteren Verlauf an Komplikationen bei Leberzirrhose.

## Diskussion

### Adenoviren

Adenoviren sind endemisch in der deutschen Gesellschaft und lassen sich auch im Tierreich (z. B. bei Hunden, Mäusen und Rindern) nachweisen [21]. Häufig treten sie in Form von Adenoviren-Konjunktivitis, aber auch Magen‑/Darminfektionen sowie als Auslöser von Atemwegserkrankungen in Erscheinung. Im Jahr 2018 wurden dem Robert Koch-Institut 676 Fälle Adenoviren-Konjunktivitis gemeldet. Im Nationalen Referenzzentraum für Influenza lag der Prozentsatz an adenoviralen Atemwegserkrankungen 2018 bei bis zu 18 % [10]. Hepatische Adenovirusinfektionen hingegen sind generell äußerst selten und hier vor allem durch die Adenovirustypen 1, 2, 5, 6, 57, 89 verursacht [10]. Aktuell wird das Adenovirus gerade als ein möglicher Auslöser vermehrter unklarer Hepatitiden bei Kindern diskutiert [9, 25]. In der Regel heilt eine Infektion mit Adenoviren im immunkompetentem Patienten folgenlos aus, Einzelfälle schwerer Verläufe sind aber beschrieben [15]. Ein fulminanter Verlauf einer Adenovirusinfektion betrifft üblicherweise immunsupprimierte Patienten, insbesondere mit hämatoonkologischen Vorerkrankungen, und verläuft dann in vielen Fällen tödlich [26]. In beiden vorliegenden Fällen zeigte sich ein prädominant hepatitisches akutes Schädigungsbild. Sehr typisch waren hierbei die diffus verteilten, fleckartigen Nekrosen, die auf keine Leberzone beschränkt waren, sowie als wichtiger Hinweis auf eine virale Genese die typischen intranukleären Einschlüsse. Der detaillierte klinische Verlauf beider Fälle wurde bereits in einer anderen Vorarbeit aufgearbeitet [11]. Differenzialdiagnostisch sollten andere infektiöse Genese wie eine Infektion durch Herpes simplex ausgeschlossen sein (z. B. mittels Immunhistochemie).

### Herpes-simplex-Virus

Eine fulminante Herpes-simplex-Virus-Hepatitis kann sowohl im Rahmen einer Reaktivierung als auch bei einer Primärinfektion auftreten, hierbei sind überwiegend immunsupprimierte Patienten betroffen, in seltenen Fällen auch scheinbar Immunkompetente [7]. Sowohl HSV‑1 und HSV‑2 können eine Hepatitis auslösen [24]. Typische orale oder genitale Läsionen betreffen nur etwa 57 % der Patienten. Die Sterblichkeit bei hepatischer Mitbeteiligung ist insgesamt hoch [13]. Histomorphologisch imponieren ebenfalls nichtzonale, fleckige Nekrosen sowie im Randbereich angrenzende Hepatozyten mit charakteristischen („Cowdry type A“) Kerneinschlüssen. Diese zeigen sich als amphophile oder eosinophile Aufhellungen mit an den Rand des Kerns gedrängtem Chromatin. Benachbarte Hepatozytenkerne können teils basophil imponieren. Differenzialdiagnostisch müssen ebenfalls andere infektiöse Genesen, insbesondere Adenoviren und Varizella zoster in Betracht gezogen werden. Je nach klinischer Konstellation (immunsupprimierende Konditionen wie Chemotherapie, Alter und hämatologische Vorerkrankungen, aber auch in immunkompetenten Patienten) können Infektionen mit HSV fulminant verlaufen, oftmals ohne typische klinische Symptome (wie z. B. orale Aphthen oder labiale Bläschen), daher sollten Leberbiopsiebefunde mit suggestiver Histologie zügig den klinischen Kollegen kommuniziert werden [29, 30]. In einem der beiden vorliegenden Fälle wurde die klinische Diagnosefindung und Therapieeinleitung erst durch die histopathologischen Befunde initiiert, da bei perakutem Infektionsverlauf, serologisch zeitlich noch nicht detektierbarem HSV-IgM und atypischer klinischer Symptomatik eine Infektion mit HSV zunächst differenzialdiagnostisch nicht weiterverfolgt wurde.

### *Coxiella burnettii*

*Coxiella burnettii* ist ein intrazelluläres Bakterium, das sich nicht durch die Gram-Färbung darstellen lässt. Es ist der Erreger des meldepflichtigen Q‑Fiebers, einer Zoonose, an der vor allem Menschen erkranken, die Kontakt mit Aerosolen von Körperflüssigkeiten (z. B. Fruchtwasser) von Schafen, Ziegen und Rindern haben. Eine Mensch-zu-Mensch-Übertragung findet bis auf berichtete Einzelfälle nicht statt [6]. Das Q‑Fieber zeigt sich klinisch mit Fieber, Pneumonie und Hepatitis, weiterhin sind auch chronische Verläufe mit Endokarditis möglich. Insgesamt ist die klinische Symptomatik häufig sehr heterogen, sodass eine Diagnosestellung meist erst mit deutlicher Verzögerung erfolgt [22]. Die Affektion der Leber durch *C. burnettii* zeigt sich häufig als granulomatöse Hepatitis mit typischen sog. Fibrinringgranulomen, einer zentralen Fettvakuole umgeben von einem Ring aus Fibrin und gemischtem entzündlichem Infiltrat. Weiterhin sind auch epitheloide Granulome sowie ausgedehnte Fibrinausschwemmungen beschrieben [18]. Es gilt zu beachten, dass insbesondere die Fibrinringgranulome nicht spezifisch für *C. burnetti* sind, sondern unter anderem auch im Rahmen von Lymphomen oder Infektionen mit Cytomegalievirus (CMV) oder den virale Hepatitiden A–E, aber auch durch Medikamente auftreten können [23]. Neben den typischen Fibrinringgranulomen sind auch histologische Bilder mit Eosinophilie oder – wie auch im uns vorliegendem Fall – florider Cholangitis beschrieben [18]. Der histologische Nachweis von Coxiellen kann mittels Giménez-Färbung erbracht werden, im Übrigen mittels PCR-Nachweis [8].

### Hepatitisviren

#### Hepatitis A

Aufgrund exzellenter Möglichkeiten zur sicheren serologischen Diagnose einer Hepatitis A sind Leberbiopsien mit akuter Hepatitis-A-Infektion inzwischen zur Ausnahme geworden. Vor allem bei drohendem akutem Leberversagen und damit Notwendigkeit zur zeitnahen Diagnosefindung werden Leberhistologie und Serologie zeitgleich gewonnen, was auch in vorliegendem Stanzzylinder der Fall war. Hepatitis A hat in Deutschland nur eine niedrige Inzidenz. In Regionen der Welt mit nur einfachen sanitären Bedingungen ist sie deutlich höher. Bei Erkrankungsfällen in Deutschland erfolgen die meisten Übertragungen innerhalb des Landes, wenn auch mehr als 30 % aller Fälle mit Auslandsreisen assoziiert sind. Der Übertragungsweg erfolgt fäkal-oral. Die Inkubationszeit ist mit 15–50 Tagen vergleichsweise lang, eine Infektiosität kann bereits bis zu 2 Wochen vor dem Auftreten von Symptomen bestehen. Eine symptomatische Hepatitis tritt in der Regel bei Erwachsenen auf, wohingegen Kinder typischerweise nur leichte oder asymptomatische Verläufe zeigen. In den meisten Fällen ist eine symptomatische Therapie ausreichend, eine Chronifizierung erfolgt nicht [5]. Nur in weniger als 1 % aller Fälle kommt es zum akuten Leberversagen [14]. Das Risiko für schwere Verläufe steigt mit dem Lebensalter und Vorerkrankungen der Leber [5]. Im hier vorliegenden Fall zeigte sich histologisch ein hepatitisches Schädigungsmuster mit lymphoplasmazellulärem Infiltrat und fleckigen, diffus zonal verteilten Nekrosen. Das Vorliegen einer Leberstanzbiopsie ist wie oben bereits erwähnt in der Regel ungewöhnlich, da in den meisten Fällen bereits eine serologische Diagnose der Hepatitis-A-Infektion erfolgt ist und somit oftmals keine Stanzbiopsie der Leber mehr angestrebt wird. Ein Fallbericht aus China zeigte, dass wohl kreuzreagierende Antikörper im Rahmen eines Overlapsyndroms von Autoimmunhepatitis und primär biliärer Cholangitis in seltenen Fällen für einen falsch positiven Nachweis von Anti-HAV-IgM verantwortlich sein können, sodass ggf. dann doch die parallel vorliegende Leberbiopsie wichtige Hinweise zur Ätiologie liefern kann [33], wobei bei Plasmazellreichtum histologisch auch die Differenzialdiagnose einer Autoimmunhepatitis in Betracht kommen kann.

#### Hepatitis E

Die Diagnose der Hepatitis E erfolgt typischerweise serologisch. Hinsichtlich des Verbreitungsweges von HEV gibt es in Abhängigkeit von den verschiedenen Serotypen deutliche globale Unterschiede. Im Gegensatz zu den endemischen Arealen in Afrika und Asien, wo eine Übertragung in den meisten Fällen über kontaminiertes Trinkwasser stattfindet und oft epidemische Ausbrüche zeigt, existieren die in Europa vorkommenden Serotypen als Zoonose. Das Reservoir stellen meistens Wildschweine dar. Der Übertragungsweg erfolgt über den Verzehr von Fleisch [12]. Der klinische Verlauf und damit auch das histologische Bild sind abhängig von den vorbestehenden Erkrankungen. Bei immunkompetenten Patienten kann es zu einer akuten Hepatitis kommen, die sich ähnlich wie eine Hepatitis-A-Infektion manifestiert, mit einem gemischtzelligem entzündlichem Infiltrat in den Portalfeldern, fleckförmigen Nekrosen, Hepatozytenballonierung, Cholestase und Cholangitis. In immunsupprimierten Patienten kommt es wie im oben beschriebenen Fall hingegen zu einer Entzündung mit chronischer Histomorphologie mit portalem mononukleärem Infiltrat mit möglicher Interface-Hepatitis, variabel ausgeprägten Hepatozytennekrosen und bei Chronifizierung auch zur Entstehung von Fibrose und Zirrhose [20]. Eine besondere Patientinnenpopulation stellen Schwangere mit akuter HEV-Infektion dar. In endemischen Regionen der Welt kommt es bei Schwangeren deutlich häufiger zum akutem Leberversagen, vor allem im dritten Trimester [16]. Hinsichtlich der Mortalität bei Schwangeren gibt es eine große Spannbreite in Abhängigkeit vom untersuchten Land. Berglov et al. berichten hierbei in ihrem systematischem Review von einer medianen „case-fatality rate“ von 26 % [2]. Ursächlich ist möglicherweise eine veränderte Expression von Toll-like-Rezeptoren [27]. Beim hier vorgestellten Fall lagen beim Patienten sowohl eine Immunsuppression als auch eine vorbestehende Siderose vor mit histologisch milden, chronisch-entzündlichen Veränderungen mit lymphozytär dominiertem Entzündungsinfiltrat in den Portalfeldern sowie der im Vergleich zum Vorbefund deutlichen progredienten Fibrose mit Ausbildung einer kompletten Zirrhose.

Zusammenfassend lässt sich feststellen, dass die Leberbiopsie bei infektiösen Ursachen der unklaren Hepatopathie einen wichtigen Beitrag zur Diagnosefindung liefern kann und bereits die Histomorphologie am HE-Schnitt sehr suggestiv sein kann. Der Einsatz zusätzlicher gewebebasierter Nachweismethoden (insbesondere Immunhistochemie, In-situ-Hybridisierung) ist hilfreich, insbesondere in den Fällen, bei denen der Nachweis von IgM-Antikörpern serologisch zeitverzögert auftreten kann (wie im oben gezeigten Fall einer fulminanten HSV-Hepatitis), oder auch in der Abgrenzung zu anderen Ursachen der unklaren Hepatopathie wie medikamentöse oder toxische Leberschädigungen [4]. An dieser Stelle sollte erwähnt werden, dass der reine immunhistochemische Nachweis auch Fallstricke birgt. So wurde kürzlich berichtet, dass in immunhistochemischen Färbungen gegen das Hepatits-E-Capsid-Protein ORF2 Kreuzreaktivität zu CMV beobachtet werden kann [19]. Infektionen mit CMV zählen wie Epstein-Barr-Virus (EBV) und Hepatitis B zu weiteren wichtigen Ursachen unklarer akuter Hepatitiden [3]. Interessanterweise spielt in diesem Kontext das Hepatitis-C-Virus (HCV) keine nennenswerte Rolle, da akute Infektionen ganz überwiegend subklinisch ablaufen und erst der Progress zur chronischen Hepatitis C klinisch manifest wird [28]. Für alle erstgenannten Erreger (CMV, EBV, HBV) existieren gute Nachweismethoden (Immunhistochemie, In-situ-Hybridisierung). Typischerweise erfolgt die Diagnose jedoch meistens serologisch, sodass Leberstanzen nur selten durchgeführt werden.

Eine Übersicht der vorgestellten Erreger als auch weiterer seltener Erreger finden Sie in Tab. 2.

**Table 2:** 

Erreger	Prädominantes histologisches Schädigungsmuster	Histologische Besonderheiten/Auffälligkeiten	Geeignete Nachweismethode
Cytomegalievirus (CMV)	Hepatitisch	Granulozytäre Mikroabszesse, virale Einschlusskörperchen („Eulenaugen-Zellen“)	Immunhistochemie
Epstein-Barr-Virus (EBV)	Hepatitisch	Selten Granulome, sinusoidale Lymphozytose („Perlschnur“)	Immunhistochemie oder In-situ-Hybridisierung
Adenovirus	Hepatitisch	Diffuse Nekrosen, virale Einschlusskörperchen	Immunhistochemie
Herpes-simplex-Virus (HSV 1 + 2)	Hepatitisch	Diffuse Nekrosen, virale Einschlusskörperchen	Immunhistochemie
Hepatitis A (HAV)	Hepatitisch	Plasmazellen (Differenzialdiagnose: Autoimmunhepatitis!), Cholestase	Klinisch (Serologie/PCR)
Hepatitis B (HBV)	Hepatitisch	Cholestase, Verlauf als fibrosierende cholestatische Hepatitis möglich	Immunhistochemie
Hepatitis E (HEV)	Hepatitisch	Cholestase, Verlauf als fibrosierende choletstatische Hepatitis möglich	Immunhistochemie
*Coxiella burnetti*/Q-Fieber	Hepatitisch oder cholangitisch	Fibrin-Ring-Granulome	Klinisch (Serologie/PCR)

## Fazit für die Praxis

- Akute Infektionen sind die zweithäufigste Ursache des akuten Leberversagens.
- Abgesehen von Hepatitis A, B und C sind andere Infektionen selten, können jedoch mitunter aufgrund charakteristischer histomorphologischer Veränderungen identifiziert werden.
- Histopathologische Kenntnis über seltene infektiöse Ursachen können die weitere Diagnostik und Therapie leiten und somit mitunter schwerwiegende Verläufe abwenden.
- Fleckförmige, diffuse, nichtzonale Nekrosen können ein wichtiger Hinweis auf ein infektiöses Geschehen sein.
